# Renoprotective Effect of the Recombinant Anti-IL-6R Fusion Proteins by Inhibiting JAK2/STAT3 Signaling Pathway in Diabetic Nephropathy

**DOI:** 10.3389/fphar.2021.681424

**Published:** 2021-05-13

**Authors:** Nanwen Zhang, Qingmei Zheng, Yaduan Wang, Juan Lin, He Wang, Rui Liu, Mengru Yan, Xiaofeng Chen, Juhua Yang, Xiaole Chen

**Affiliations:** ^1^ School of Pharmacy, Department of Pharmacology, Fujian Medical University, Fuzhou, China; ^2^ Fujian Key Laboratory of Drug Target Discovery and Structural and Functional Research, School of Pharmacy, Fujian Medical University, Fuzhou, China; ^3^ School of Pharmacy, Department of Bioengineering and Biopharmaceutics, Fujian Medical University, Fuzhou, China; ^4^ School of Integrative Medicine, Fujian University of Traditional Chinese Medicine, Fuzhou, China; ^5^ Rehabilitation Hospital Affiliated to Fujian University of Traditional Chinese Medicine, Fuzhou, China

**Keywords:** diabetic nephropathy, IL-6, GK rat, STZ, JAK2/STAT3

## Abstract

Diabetic nephropathy the main reason for end stage renal disease is a common microvascular complication in patients with type 1 and type 2 diabetes. The interleukin-6 (IL-6), acting as a pleiotropic cytokine, play key roles in main autoimmune disorders. The recombinant anti-IL-6R fusion proteins (VHH-0031) constructed and obtained in our lab is a dual target-directed single domain-based fusion protein against the interleukin-6 receptor. This study aims to explore the renoprotective effect of VHH-0031 in diabetic nephropathy. VHH-0031 treatment alleviated renal inflammation, morphologic injury and renal insufficiency in both Goto-Kakizaki rats and STZ-induced Sprague Dawley rats. These renoprotective effects of VHH-0031 are associated with alleviating inflammation and suppression of the JAK2/STAT3 signaling pathway. The mesangial cells treated with VHH-0031 exhibited anti-proliferation, anti-inflammation and inactivation of JAK2/STAT3 pathway under high glucose condition. In conclusion, this study demonstrates that VHH-0031 exhibited a potent protective effect in kidney of diabetic rats and its mechanism may be concerned with the inhibition of the IL-6R/JAK2/STAT3 pathway of glomerular mesangial cells.

## Introduction

Diabetic nephropathy (DN) is a common microvascular complication in either patients with type 1 or type 2 diabetes (Xu et al., 2018). At present, besides strict control of blood sugar, the main treatment methods of DN include angiotensin converting enzyme inhibitor (ACEI) and angiotensin II receptor blocker (ARB) (Lv et al., 2012) dipeptidyl peptidase-4 (DPP-4) inhibitors, and Sodium glucose co-transporter-2 (SGLT-2) inhibitors, mineralocorticoid receptor antagonism and endothelin antagonists have been shown to retard DN in a large randomized trial (Heerspink et al., 2016; Heerspink et al., 2019; Bakris et al., 2020). Losartan, the first angiotensin receptor blocker (ARB) and the ACE inhibitor captopril, is commonly used in clinical trials (Messerli et al., 2018). Linagliptin is a selective DPP-4 inhibitor employed for glycemic management of type 2 diabetes (Rosenstock et al., 2019). SGLT2 inhibitors, including dapagliflozin, canagliflozin and empagliflozin are used to approve anti-hyperglycemic therapies (Wanner et al., 2016; Perkovic et al., 2019; Wheeler et al., 2021). Finerenone, a nonsteroidal selective mineralocorticoid receptor antagonist, reduced albuminuria in short-term trials involving patients with chronic kidney disease (CKD) and type 2 diabetes (Bakris et al., 2020). Atrasentan is the endothelin receptor antagonist in protecting renal function in patients with type 2 diabetes at high risk of developing end-stage kidney diseases (Heerspink et al., 2019). All the medication mentioned above are now widely accepted anti-hyperglycemic therapies. Though these drugs can only partially address the problem, they still slow the progression of DN rather than prevent. Meanwhile, they have to do with the increase in adverse events such as lowering of blood pressure, decreased oxygen demand (Emdin et al., 2015; Majewski and Bakris, 2016; Thomas et al., 2017). The research on treatment of diabetic nephropathy has representative and guiding significance.

The pathogenesis of DN is diverse, such as hemodynamic changes, metabolic disorders, oxidative stress, inflammation (Lim, 2014). Many studies have reported that the inflammation mechanism has been accepted as the central factor to promote the development of DN (Donate-Correa et al., 2015; Sun and Kanwar, 2015). DN is characterized by enhanced level of pro-inflammatory cytokines such as IL-6, TNF-α and IL-1β (Feigerlová and Battaglia-Hsu, 2017). IL-6 is considered to be the driving signal of many inflammatory and lymphoproliferative diseases, such as rheumatoid arthritis and Castleman's disease, and several therapies have been developed to target IL-6 signaling pathway (Garbers et al., 2018). A large number of studies support the role of IL-6 signal transduction in the development of DN. For example, the level of serum IL-6 in DN patients is higher than that without DN patients (Taslipinar et al., 2011) and the risk of DN in type 2 diabetes mellitus (T2DM) patients with high IL-6 gene polymorphism is increased (Papaoikonomou et al., 2013). Therefore, IL-6 could be a potential target to provide new therapeutic strategies to improve the treatment of DN.

Due to the relatively large size and poor stability, affecting the route of use, and high cost, conventional antibody drugs have greatly limited the clinical use of them (Omidfar et al., 2013; Farajpour et al., 2014). The naturally occurring heavy chain antibodies (HcAb’s) lacking light chain and CH1 domains, are composed of a single domain called VHH or nanobodies (Tu et al., 2020), which have been described as the second type of antibodies. The characteristics of VHH include heat resistance, small size, high solubility, low immunogenicity, and the ability of refolding after denaturation, maintain the binding ability, which makes these antigen-specific antibody fragments have a remarkable prospect in clinical application. In addition, they also have a long region of antigen recognition, which enhances their specific ability to target antigens (Bannas et al., 2017). The recombinant anti-IL-6R fusion proteins (VHH-0031), by linking 2 single domain chains against the pro-inflammatory cytokine receptor (IL-6R) and human serum albumin (HSA) respectively, was constructed successfully and obtained by our developed prokaryotic expression system. The single domain chains against HSA have been reported not only significantly increase the half-life of antibodies *in vivo*, but also selectively direct the antibodies to specific organs. The present study investigated whether VHH-0031 could improve the progression of DN in either GK rats (T2DM) or streptozotocin-induced SD rats (T1DM) and elucidate its underlying mechanism.

## Material and Methods

### Animals

24 Goto-Kakizaki (GK) rats (6–8 weeks, 160–180 g, male) and 6 Wistar rats (6–8 weeks, 160–180 g, male) were purchased from Changzhou Cavens Laboratory Animal Co., Ltd. After 1 week for acclimation, rats were randomly divided into five groups (n = 6 per group): 1) Control group: Wistar rats; 2) Model group: GK rats; 3) Metformin group (model + Metformin 500 mg kg^−1^); 4) VHH-0031 0.1 mg kg^−1^ (model + recombinant anti-IL-6R fusion proteins 0.1 mg kg^−1^); (5)VHH-0031 0.5 mg kg^−1^ (model + recombinant anti-IL-6R fusion proteins 0.5 mg kg^−1^). The GK rats were induced with high fat diet and met the model standard (random blood sugar was higher than 11.1 mmol.L^−1^). The recombinant anti-IL-6R fusion proteins (VHH-0031) and Metformin were injected intraperitoneally once per three days for 7 weeks while the control group and the model group received an equivalent volume of distilled water.

30 Sprague Dawley (SD) rats (6–8 weeks, 160–180 g, male) were purchased form Laboratory Animal Center of Fujian Medical University. After 1 week for acclimation, rats were randomly divided into five groups (n = 6 per group): 1) Control group: SD rats without STZ treatment; 2) Model group: SD rats with STZ treatment; 3) Benazepril group (model + benazepril 10 mg kg^−1^); 4) VHH-0031 0.1 mg kg^−1^ (model + recombinant anti-IL-6R fusion proteins 0.1 mg kg^−1^); 5) VHH-0031 0.5 mg kg^−1^ (model + recombinant anti-IL-6R fusion proteins 0.5 mg kg^−1^). The model group was injected with STZ 30 mg kg^−1^·day for 3 days intraperitoneally to establish the type 1 diabetic model, and the control group was injected with the same amount of citric acid buffer. One week after STZ injection, the blood glucose of rats was measured. The rats with blood glucose over 16.7 mmol L^−1^ were successfully modeled and chosen for follow-up study. The recombinant anti-IL-6R fusion proteins (VHH-0031) and Benazepril were injected intraperitoneally once per three days for 6 weeks while the control group and the model group received an equivalent volume of distilled water.

The animals were kept under standard laboratory conditions with a temperature of 21 ± 2°C, a relative humidity of 55%, and a 12 h:12 h light-dark cycle. At the end of the experiment, rats were euthanized with 2% sodium pentobarbital by intraperitoneal injection. After anesthesia, blood was taken from the rat veins and left at room temperature for 2 h, centrifuged at 2500 × *g* for 20 min, the supernatant was collected and stored for use. After blood collection, the kidney samples of rats were taken out and weighed. The Viscera index was calculated for pathological examination and tissue biochemical index determination. Part of the kidney tissue was fixed in 4% paraformaldehyde, and the rest was stored at −80°C for biochemical analysis. All procedures were performed in accordance with protocols approved by the Ethics Review Committee for Animal Experimentation of Fujian Medical University (No. 2018-108). All animals were raised in Laboratory Animal Center of Fujian Medical University (Certificate No. SYXK (Fujian) 2016-0006), where the animal work has taken place, and animal handling procedures were performed in strict accordance with the care of laboratory animals according to the Fujian Province Zoological Society.

### Determination of Random-Blood Glucose, Serum Creatinine, Serum Urea Nitrogen

The weight of rats was measured weekly, and the blood sugar was measured by blood glucose meter (J & J). Serum creatinine and blood urea nitrogen (Nanjing Jiancheng Bioengineering Institute) were determined by biochemical kit according to the introduction.

### Enzyme-Linked Immunosorbent Assay

For the determination of TNF-α and IL-6 concentration in serum, an enzyme-linked immunosorbent assay (ELISA) kit from Wuhan Boshide Biological Engineering Co. Ltd. was used. Assay procedures were as described in the product manual, using the internal standard calibration curve to calculate the results. Optical density (OD) of the samples was measured at a wavelength of 405 nm using a microplate reader (Thermo, United States of America).

### Histopathology

For histological analysis, 4% paraformaldehyde-fixed kidney tissues were dehydrated through a graded series of ethanol, routine paraffin embedding, and then cut into 3 μm sections to make tissue slides. After dewaxing and hydration, Hematoxylin and Eosin, Periodic acid-schiff (PAS) and Masson staining were used to observe glomerular morphology changes and collagen deposition. The morphology of kidney tissue was observed by optical microscopy and magnified with a high-resolution camera ×400 images. The mesangial matrix index was quantified using Image-Pro Plus 6.0 (Media Cybernetics, Silverspring, MD, United States of America)software by PAS staining in 10 random fields ( ×400) as previously described (Glastras et al., 2016). The glomerulosclerosis was quantified by Masson’s trichrome staining, where 20 glomeruli were randomly selected from each section and positive signals within the selected glomerulus were highlighted, measured, and represented as percentage positive area of the entire glomerulus.

### Immunohistochemistry

After the kidney tissue sections were dewaxed with ascites, they were treated with 3% H_2_O_2_ for 10 min, PBS containing 1% BSA was added for 30 min, and incubated with JAK2 (Abcam, Cat#108596), STAT3(Cell Signaling Technology, Cat#12640) diluted for 1:400 at 4°C overnight. The slides were then incubated with goat anti-rabbit polyperoxidase without biotin. After incubation for 30 min, immunostaining was performed with 0.05% diaminobenzene. The section was examined with an optical microscope (Leica) and magnified with a high-resolution camera ×400 images. The positive staining area and the

Glomerular tuft area were calculated in 20 glomeruli per rat with Image-Pro Plus 6.0, and the stained area was expressed as a percentage of the glomerular tuft area.

### Real Time Quantitative PCR

Total RNA was extracted with RNA kit and reverse transcription into cDNA according to the instructions. Real time quantitative PCR was performed using SYBR green master mix (Roche, Germany) in a Roche Light cycler 96 system. 2^−ΔΔCt^ method was used to calculate the relative mRNA expression level. All experiments described were performed at least 3 times. Results are presented as ratio of mRNA level, taking the control as 1. The primers used in RT-PCR were GAPDH (Forward,5′-CTAGTGGAGTCTACTGGTGT-3′; Reverse,5′-GTCATCATACTTGGCAGGTT-3′),TNF-α(Forward,5′-GCACAGCCTTCCTCACAGAG-3′;Reverse,5′-ACCCGTAGGGCCATTACAGT-3′),IL-6(Forward,5′-GGGGCAAGCCTTCCAGTTAG-3′;Reverse,5′-CTCGAGAGAGACCCATGCCT-3′),JAK2.

(Forward,5′-GGTTCATTCAGCAGTTCAGTC-3′; Reverse,5′-GCAGGGTCTCCAGGTTTATG-3′),STAT3(Forward,5′-TACCACAAAAGTCAGGTTGCTG-3′; Reverse,5′-ACATCCCCAGAGTCCTTATCAA-3′),TGF-β1(Forward,5′-AGGGCTACCATGCCAACTTC-3′; Reverse,5′-AGGGCTACCATGCCAACTTC-3′),CyclinD1(Forward,5′-CTGACAACTCTATCCGCCC-3′; Reverse,5′-CATCCGCCTCTGGCATTTTG-3′)

### Cell Culture

Rat renal mesangial cells (HBZY-1, Shanghai Fuheng Biology, China) were cultured in DMEM medium (Gibco, United States of America) with 5.5 mmol L^−1^ glucose. When the cells adhere to the wall to 80% fusion, cells were digested the passage with trypsin. The cells were cultured at 37°C in 5% CO_2_ saturated humidity incubator.

### Cell Proliferation Analysis

HBZY-1 cells were seeded in 96-well plates at a density of 1 × 10^5^ cells·ml^−1^. After 24 h of adherence, the cells were cultured with serum-free DMEM medium for 24 h to synchronize. Next, cells were cultured under a condition of normal glucose (NG, 5.5 mM D-glucose), high glucose (HG, 25 mM D-glucose) and high glucose (HG, 25 mM D-glucose) accompany with the recombinant anti-IL-6R fusion proteins for 48 h. Then MTT assay was used to measure cell proliferation activity according to the manufacturer’s instructions.

### Measurement of Cell Cycle

The cells were collected and fixed with 70% cold ethanol (2 h to overnight) and added with PI/RNase (KGA511-KGA512) a dye working solution prepared in advance. Then cells were kept away from light for 30–60 min at room temperature. The red fluorescence at 488 nm was recorded. The proportion of each cell cycle was analyzed.

### Western Blot

The cells were harvested and lysed with RIPA lysate. Lysate samples were separated by SDS-PAGE and transferred to PVDF membrane. The membrane was sealed with 5% skim milk powder at room temperature, and then incubated with β-tubulin (Cell Signaling Technology, Cat#2128), JAK2 (Abcam, Cat#108596), STAT3 (Cell Signaling Technology, Cat#12640), P-STAT3 (Cell Signaling Technology, Cat#9145), Cyclin D1 (Cell Signaling Technology, Cat#55506), Cyclin E1 (Cell Signaling Technology, Cat#20808). Then it was incubated with a secondary antibody binding to horseradish peroxidase and visualized the immune response zone with enhanced chemiluminescence reagent. All recommend antibody dilutions are 1:1,000.

### siRNA Interference

HBZY-1 cells were seeded in six-well plates under high glucose condition. Cells were transfected with IL-6siRNA (Forward,5′-GAUGAUGCACUGUCAGAAAdTd-3′; Reverse,5′-UUUCUGACAGUGCAUCAUCdTd-3′), siRNA-scramble (Forward, 5′-UUC UCCGAACGUGUCACGUdTd-3′;Reverse,5′-ACGUGACACGUUCGGAGAAdTd-3′) and the transfected concentration is 2 pmol/well (Han Heng biology, China) list for 8 h and then treated with the recombinant anti-IL-6R fusion proteins (VHH-0031 5 pM) for 48 h. The cells were then harvested for mRNA analysis.

### Statistical Analysis

All results are expressed as mean ± SD. Data were analyzed using analysis of variance (ANOVA), followed by post hoc Bonferroni tests (GraphPad Prism 8.0, GraphPad Software, San Diego, CA, United States of America). A *p* value of <0.05 was considered statistically significant. In addition, a repeated analysis ANOVA analysis was used for body weight, fasting blood glucose analysis.

## Results

### Effect of VHH-0031 Prevents the DN in GK Rats

**FIGURE 1:**
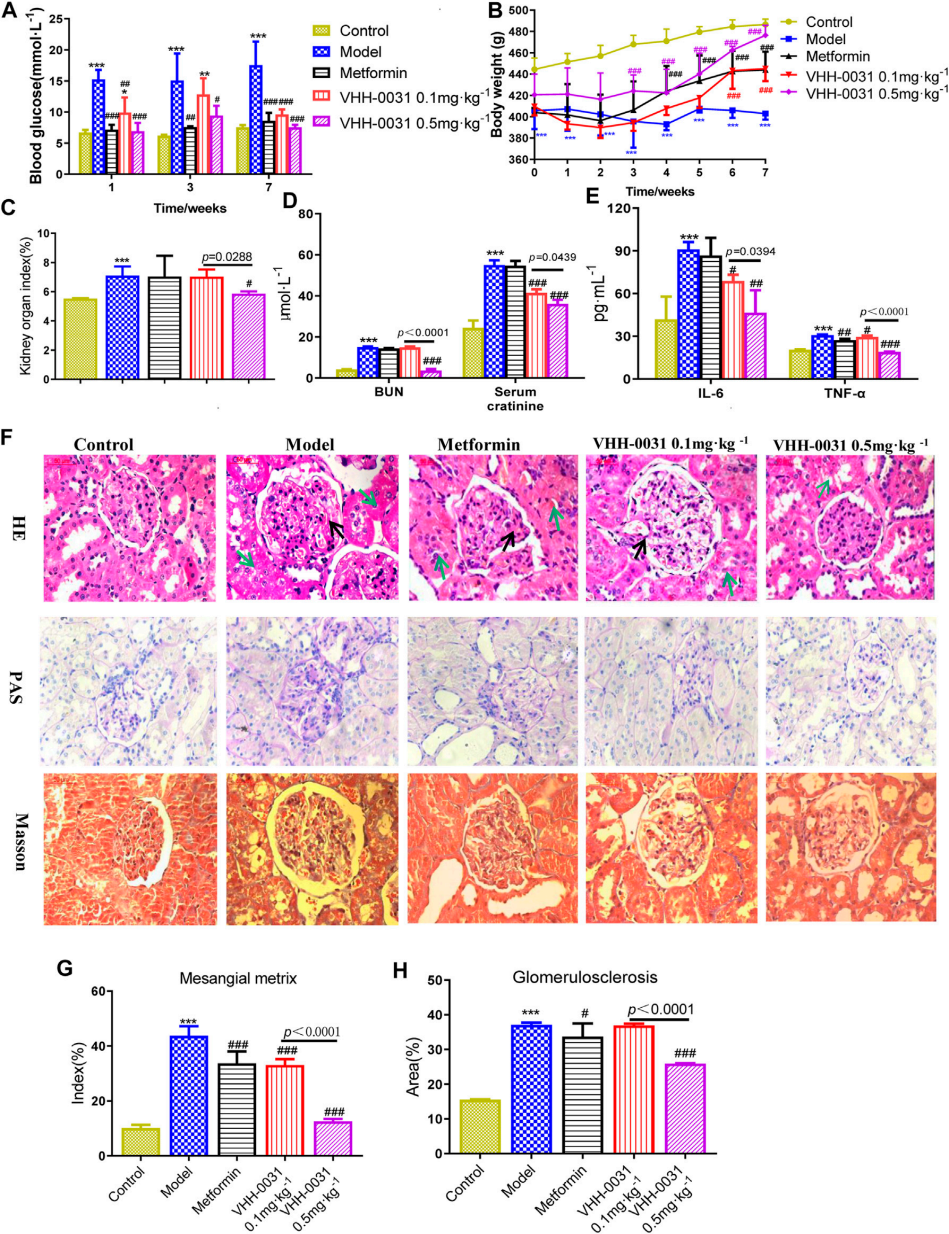
The DN prevention in GK rats by VHH-0031. Rats were injected with saline, metformin (500 mg kg^−1^), VHH-0031 (0.1 or 0.5 mg kg^−1^) for 7 weeks, and then killed for study **(A)** Blood glucose level, **(B)** Body weight change **(C)** The ratio of kidney weight to body weight **(D)** The level of blood urea nitrogen and serum creatinine in the serum by experimental kit **(E)** The level of IL-6 and TNF-α in the serum by ELISA **(F)** Evaluation of mesangial expansion, mesangial metrix caused by glycogen stimulation and glomerulosclerosis caused by collagen deposition in the kidney by H&E, PAS, Masson staining, Bar = 100 μm. The black arrow points to glomerular mesangial hyperplasia, the green arrow points to renal tubule congestion and edema. Quantification analysis of mesangial matrix index by PAS staining **(G)** and glomerulosclerosis by Masson’s trichrome staining **(H)** in kidneys. Control: Wistar rats, Model: GK rats, Metformin: GK rats treated with metformin 500 mg kg^−1^, VHH-0031 0.1 and 0.5 mg kg^−1^: GK rats treated with the recombinant anti-IL-6R fusion proteins (0.1 and 0.5 mg kg^−1^). Data represents the mean ± SD for six rats per group. *p < 0.05, **p < 0.01, ***p < 0.001 *vs* control group. ^#^ *p* < 0.05, ^##^ *p* < 0.01, ^###^ *p* < 0.001 *vs* model group.

GK diabetic model was employed to evaluate the therapeutic effect of VHH-0031 (recombinant anti-IL-6R fusion proteins) in T2DM related kidney injury. The model GK rats had remarkable higher blood glucose levels than the control Wistar rats, compared with the model group, blood glucose levels were reduced after VHH-0031 treatment, and the difference was statistically significant (Figure 1A). The application of VHH-0031 was able to reduce the rise of blood sugar and hinder weight loss in GK rats. Following, we focused on kidney functions and evaluated the potential renoprotection effects of VHH-0031. VHH-0031 treatment increased body weight (Figure 1B) and reversed kidney/body weight ratio (Figure 1C) in GK rats. The serum urea nitrogen and creatinine levels were upregulated in renal injury. As shown in Figure 1D, the level of blood urea nitrogen and serum creatinine in GK rats were higher compared with control Wistar rats. However, the elevated blood urea nitrogen and serum creatinine parameters in GK rats could be reversed in VHH-0031-treated GK rats.

Inflammatory cytokines are believed to be involved in the progression of DN, thus we explored whether the improvement of renal function and glucose metabolism in diabetic nephropathy rats was accompanied by the change of these cytokines. As expected, after administration of VHH-0031, the increased IL-6 and TNF-α in serum were dramatically reduced (Figure 1E). H&E, PAS, and Masson's trichrome staining were employed to evaluate the changes of renal histopathology. The results showed that the renal tissue of GK stained by H & E had the characteristics of glomerular structure fuzziness, glomerular adhesion, glomerular hypertrophy and mesangial hyperplasia, but not significantly changed in control Wistar rats. More importantly, VHH-0031 significantly suppressed renal histological injury including glomerular matrix deposition and glomerulosclerosis (Figures 1F–H).

**FIGURE 2:**
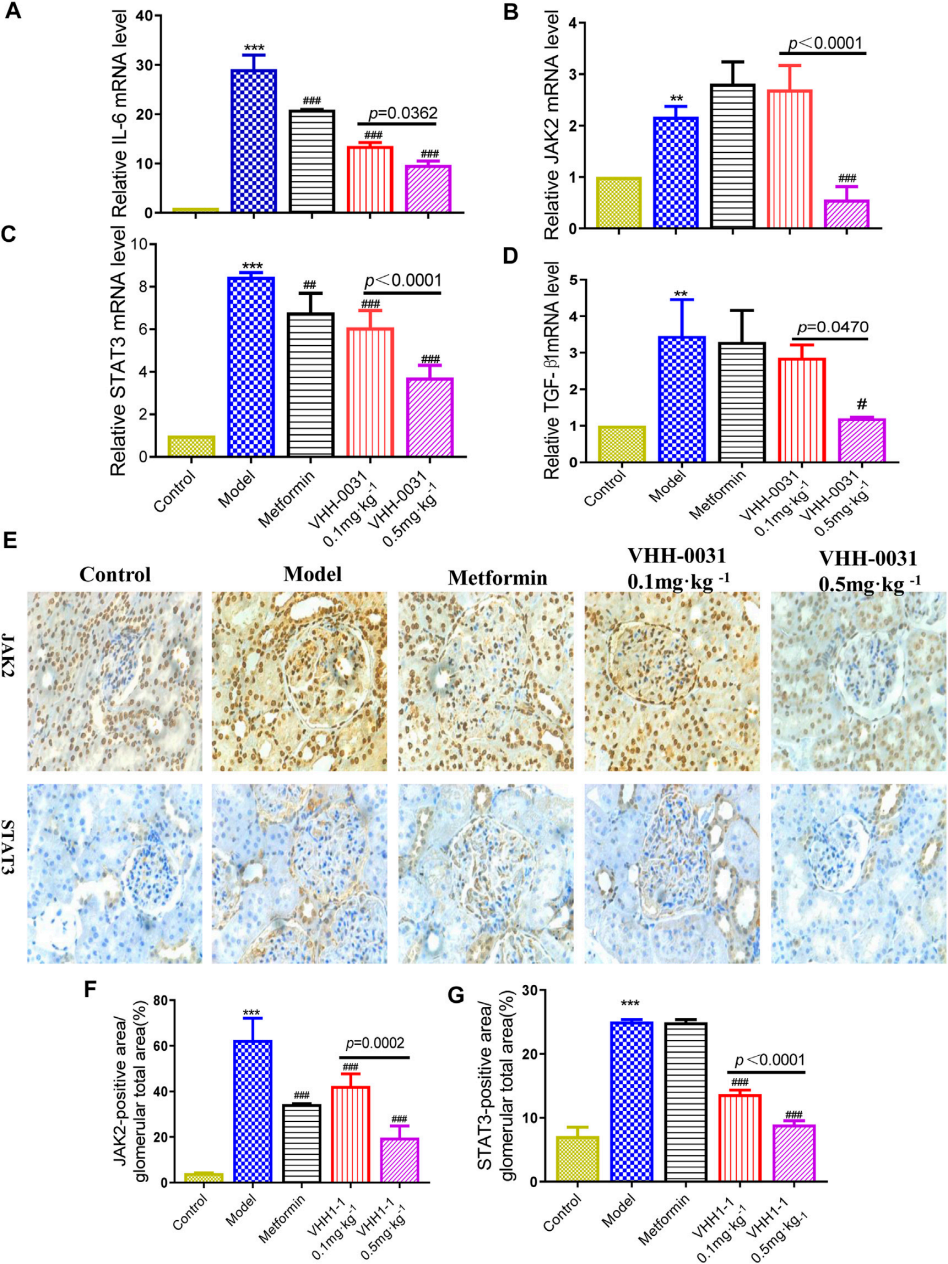
Effect of VHH-0031 on JAK2-STAT3 signaling pathway in GK rats kidney. The mRNA expression of IL-6 **(A)**, JAK2 **(B)**, STAT3 **(C)** and TGF-β1 **(D)** in kidney determined by qRT-PCR. The expression of JAK2 and STAT3 staining measured by immunohistochemistry in kidney **(E)**, Bar = 100 μm ( ×400). Quantification of JAK2 **(F)** and STAT3 **(G)** protein measured by immunohistochemical staining. Data represents the mean ± SD for six rats per group. Control: Wistar rats, Model: GK rats, Metformin: GK rats treated with metformin 500 mg kg^−1^, VHH-0031 0.1 and 0.5 mg kg^−1^:GK rats treated with the recombinant anti-IL-6R fusion proteins (0.1 and 0.5 mg kg^−1^). *p < 0.05, **p < 0.01, ***p < 0.001 *vs* control group. ^#^ *p* < 0.05, ^##^ *p* < 0.01, ^###^ *p* < 0.001 *vs* model group.

In the glomerular sclerosis and matrix deposition of diabetic nephropathy, JAK2/STAT3 is the most important signal cascade involved in IL-6 transduction. Similarly, they are also important substances in the inflammatory process of IL-6. To further confirm the effects of VHH-0031, qRT-PCR was employed for analyzing their mRNA level. Compared with control Wistar rats, the mRNA level of IL-6, JAK2, STAT3 and TGF-β1 increased significantly in GK rats, though VHH-0031 intervention could reverse these increases ((Figures 2A–D)). Thus, we observed the effect of VHH-0031 on the expression of JAK2/STAT3 expression in diabetic nephropathy. Based on the results of immunohistochemistry, compared with control, the protein expression of JAK2 and STAT3 in the renal tissue of GK rats was significantly increased, mainly in mesangial cells and podocytes of the glomerulus. VHH-0031 treatment led to a remarkable decrease of JAK2 and STAT3 protein ((Figures 2E–G)).

### Effect of VHH-0031 Prevents the DN in STZ-Induced Diabetes Rats

**FIGURE 3:**
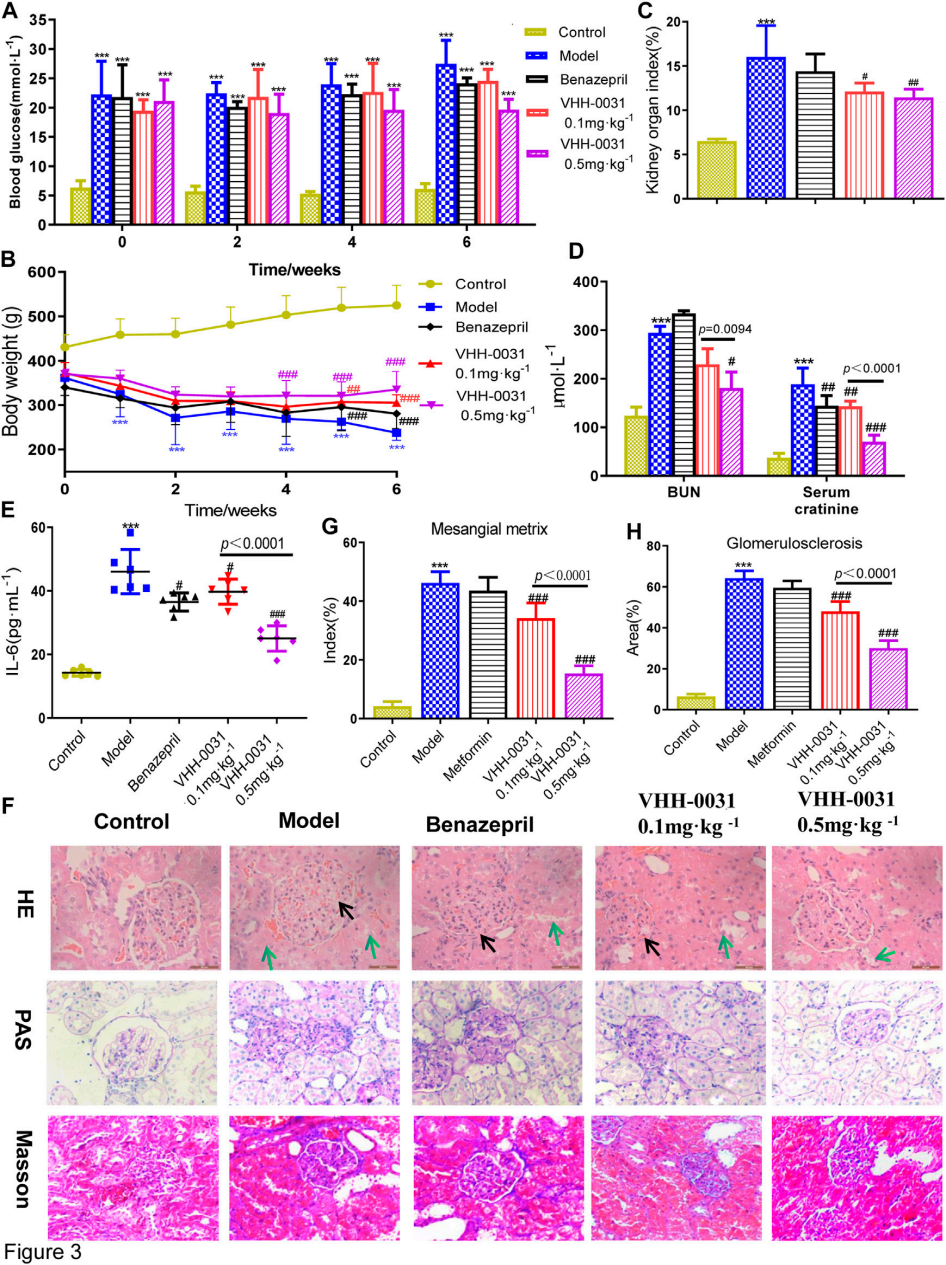
The DN prevention in STZ-induced rats by VHH-0031. Rats were administrated with saline, benazepril (10 mg kg^−1^), VHH-0031 (0.1 or 0.5 mg kg^−1^) for 6 weeks, and then killed for study **(A)** Blood glucose level **(B)** Body weight change **(C)** The ratio of kidney weight to body weight **(D)** The level of blood urea nitrogenand serum creatinine in the serum by experimental kit **(E)** The level of IL-6 in the serum by ELISA **(F)** Evaluation of mesangial expansion, mesangial metrix caused by glycogen stimulation and glomerulosclerosis caused by collagen deposition in the kidney by H&E, PAS, Masson staining, Bar = 100 μm.The black arrow points to glomerular mesangial hyperplasia, the green arrow points to renal tubule congestion and edema. Quantification analysis of mesangial matrix index by PAS staining **(G)** and glomerulosclerosis by Masson’s trichrome staining **(H)** in kidneys Bar = 100 μm. Data represents the mean ± SD for six rats per group. Control: SD rats without STZ treatment, Model: SD rats with STZ treatment, Benazepril 10 mg kg^−1^: SD rats with STZ treatment in presence of benazepril (10 mg kg^−1^), VHH-0031 0.1 and 0.5 mg kg^−1^: SD rats with STZ treatment in presence of the recombinant anti-IL-6R fusion proteins (0.1 and 0.5 mg kg^−1^). **p < 0.01, ***p < 0.001 *vs* control group, ^#^ *p* < 0.05, ^##^ *p* < 0.01, ^###^ *p* < 0.001 *vs* model group.

STZ-induced diabetes rats were used to study the improvement effect of VHH-0031 on type 1 related diabetic nephropathy. The results showed that blood glucose increased sharply in STZ-induced rats, however, VHH-0031 intervention did not lower blood glucose well, only maintained blood glucose without letting it worsen in the STZ-induced rats (Figure 3A). In VHH-0031 treatment group, body weight began to rise slowly from the second week, however it was slower than that of GK rats (Figure 3B). Similar to GK rats, VHH-0031 treatment could decrease STZ-induced increased kidney/body weight ratio (Figure 3C), the level of blood urea nitrogen, serum creatinine (Figure 3D) and IL-6 (Figure 3E).

The histological examination was also employed to observe the pathological changes of kidney in STZ-induced diabetic rats. The Figure 3F showed that glomerulus had obvious pathological changes (HE staining), accompanied by glomerular matrix deposition (PAS staining) and glomerulosclerosis caused by collagen deposition (Masson's trichrome staining) accumulation. VHH-0031 treatment could reduce pathological changes caused by STZ-induced diabetes in (Figures 3F–H).

**FIGURE 4:**
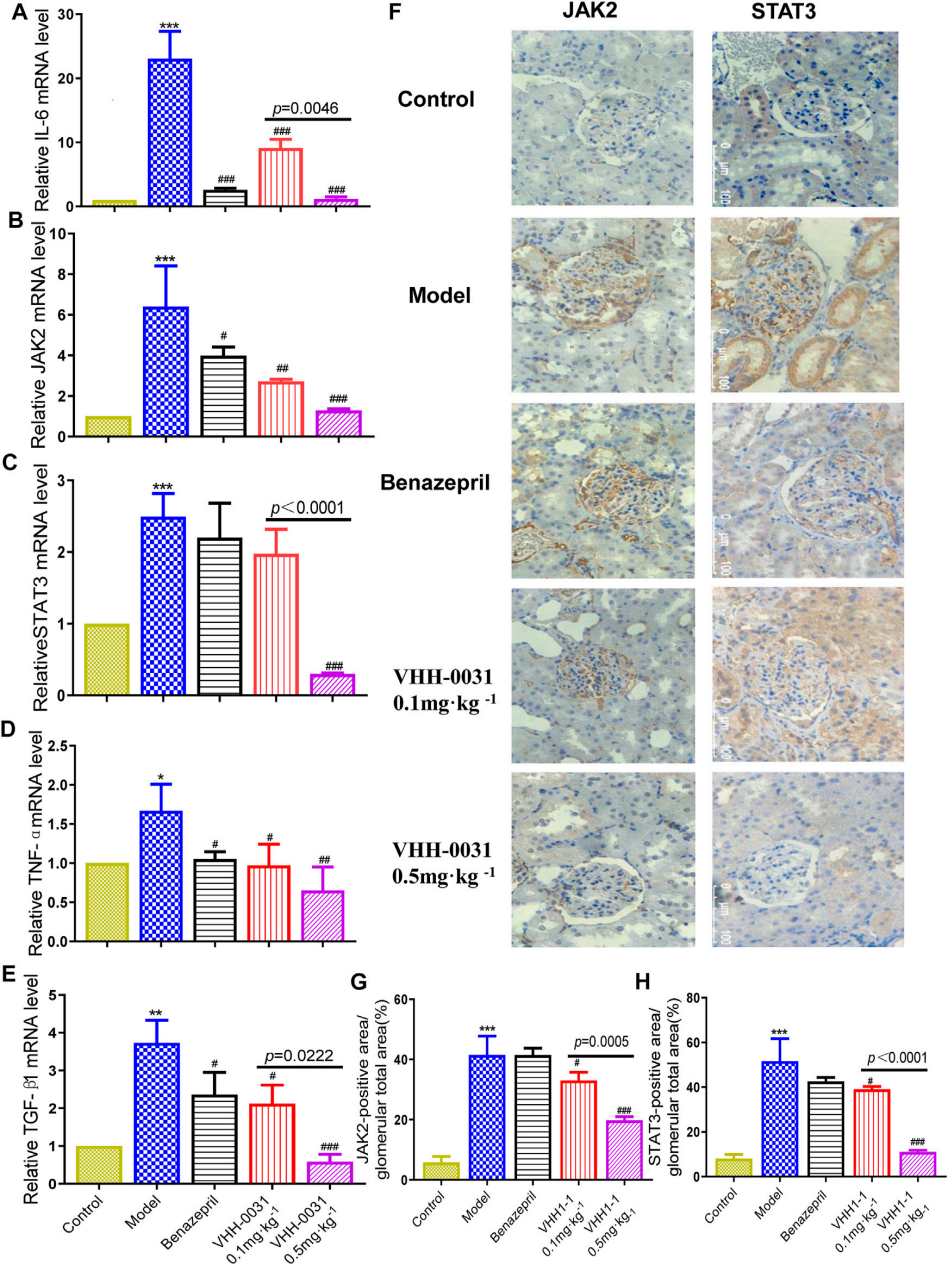
Effect of VHH-0031 on JAK2-STAT3 signaling pathway in STZ-induced rats kidney. The mRNA expression of IL-6 **(A)**, JAK2 **(B)**, STAT3 **(C)**,TNF-α **(D)** and TGF-β1 **(E)** in kidney determined by qRT-PCR **(F)** The expression of JAK2 and STAT3 staining measured by immunohistochemistry in kidney,Bar = 100 μm ( ×400). Quantification of JAK2 **(G)** and STAT3 **(H)** protein measured by immunohistochemical staining. Data represents the mean ± SD for six rats per group. Control: SD rats without STZ treatment, Model: SD rats with STZ treatment, Benazepril 10 mg kg^−1^: SD rats with STZ treatment in presence of benazepril (10 mg kg^−1^), VHH-0031 0.1 and 0.5 mg kg^−1^: SD rats with STZ treatment in presence of the recombinant anti-IL-6R fusion proteins (0.1 and 0.5 mg kg^−1^). **p < 0.01, ***p < 0.001 *vs* control group, ^#^ *p* < 0.05, ^##^ *p* < 0.01, ^###^ *p* < 0.001 *vs* model group.

Results consistent with GK rats, the increased mRNA level of such as IL-6 (Figure 4A), JAK2 (Figure 4B), STAT3 (Figure 4C), TNF-α (Figure 4D) and TGF-β1 (Figure 4E) were observed in the kidney tissue of STZ-induced diabetic rats, where they were reversed by VHH-0031 treatment. Meanwhile, the immunohistochemistry and qRT-PCR were used to explore related signal pathway in kidney tissue of STZ-induced rats. The results showed similar increased expression of JAK2 and STAT3 in the kidney tissue of STZ-induced rats with that in GK rats, but in VHH-0031-treated group, these changes were significantly attenuated ((Figures 4F–H)).

### Effect of VHH-0031 Prevents Proliferation of Mesangial Cells Under High Glucose Condition

**FIGURE 5:**
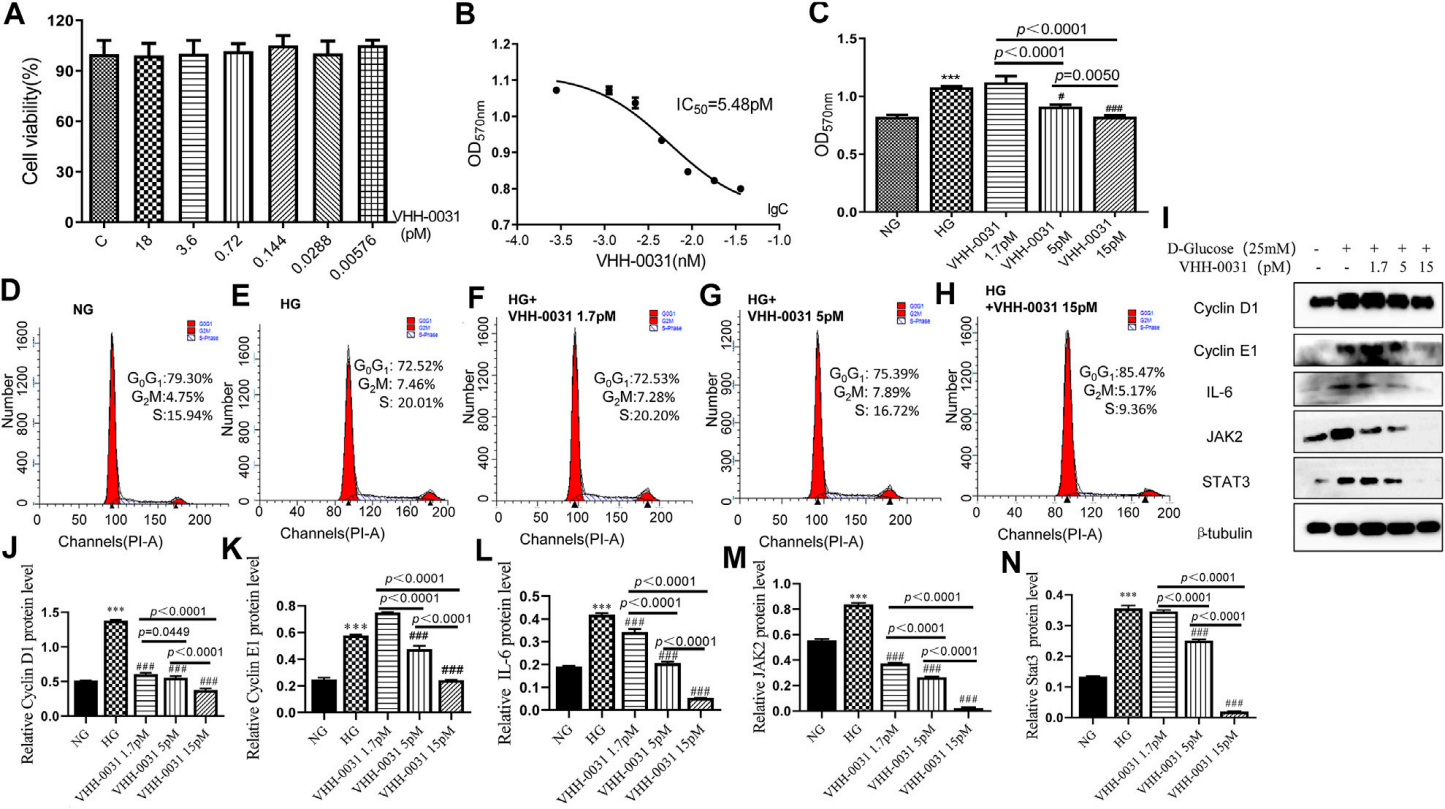
VHH-0031 prevents proliferation of mesangial cells under high glucose condition *via* JAK2/STAT3 pathway **(A)** MTT method was performed to measure the cell viability after incubation of different doses of VHH-0031 (0∼18pM) in condition of normal glucose (NG, 5.5 mM D-glucose) **(B,C)** Detection of cell proliferation by MTT. Cells cycle were measured by flow cytometry. Cells were treated with **(D)** normal glucose (NG, 5.5 mM D-glucose) **(E)** high glucose (HG, 25 mM D-glucose), or **(F–H)** high glucose (HG, 25 mM D-glucose) in the presence of VHH-0031 (1.7, 5, and 15 pM) for 48 h (I)Western blot were employed to detected the protein of Cyclin D1, Cyclin E1, IL-6, JAK2 and STAT3. Quantitative analysis of Cyclin D1 **(J)**, Cyclin E1 **(K)**, IL-6 **(L)**, JAK2 **(M)**, and STAT3 **(N)** in HBZY-1. ***p < 0.001 *vs* NG group. ^###^ *p* < 0.001 *vs* HG group.

Since mesangial cells play key roles in the development of DN, high-glucose-induced mesangial cells have been chosen to investigate the activity of VHH-0031 *in vitro*. We explored the cytotoxicity of VHH-0031 on mesangial cells, the cells were handled with VHH-0031 (0–18 pM) for 48 h. MTT result showed no remarkable changes in cell viability after VHH-0031 treatment (Figure 5A). The mesangial cells were following treated with or without VHH-0031 in high glucose condition for 48 h. The results showed that high glucose challenge leaded to a significant increase of cells proliferation. After incubation of VHH-0031, high-glucose-induced cell proliferation were inhibited with IC_50_ of 5.48 pM (Figure 5B). According to the pre-experiment, three concentrations of 1.7, 5, and 15 pM were selected for subsequent experiments (Figure 5C).

Cell cycle is a key mechanism to control cell proliferation. We detected the cell cycle of mesangial cells by flow cytometry. Results as shown in (Figures 5D–H), under the condition of high glucose, the cell number of G_0_/G_1_ phase reduced, while that of S phase and G_2_/M phase elevated dramatically. VHH-0031 treatment dose-dependently reversed changes of cell number in each phase induced by high glucose, caused cell cycle arrest in G_0_/G_1_ phase and inhibited cell proliferation. In addition, VHH-0031 treatment attenuated high-glucose-induced increase expression of cyclin D1 (Figure 5J), cyclin E1 (Figure 5K), IL-6 (Figure 5L), JAK2 (Figure 5M), and STAT3 (Figure 5N) according to Western blot (Figure 5I) analysis.

### Effect of VHH-0031 Suppresses JAK2/STAT3 Pathway in High-Glucose and IL-6 siRNA Stimulated Mesangial Cells

**FIGURE 6:**
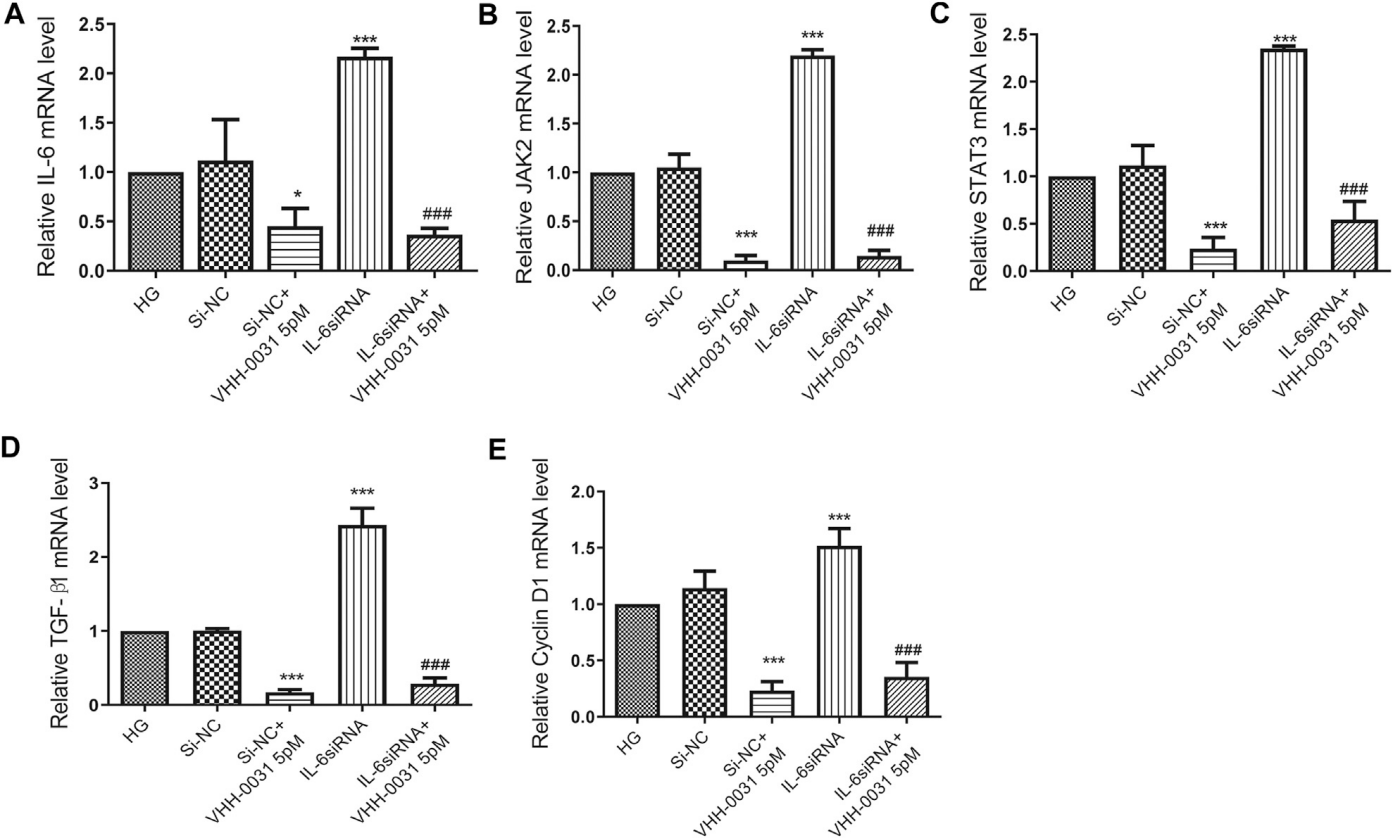
Effect of VHH-0031 suppresses JAK2/STAT3 pathway in high-glucose and IL-6siRNA stimulated mesangial cells. Cells were treated with random sequence IL-6siRNA (Si-NC), or random sequence IL-6siRNA and VHH-0031(Si-NC + VHH-0031 5pM), or IL-6siRNA, or IL-6siRNA and VHH-0031 (IL-6siRNA + VHH-0031 5 pM) in the presence of high glucose (HG). The mRNA expression level of IL-6 **(A)**, JAK2 **(B)**, STAT3 **(C)**, TGF-β1 **(D)** and Cyclin D1 **(E)** in HBZY-1 cells were detected by qRT-PCR. *p < 0.05, ***p < 0.001 *vs* HG group, ^###^ *p* < 0.001 *vs* IL-6siRNA group.

JAK2 and STAT3, playing key roles in the control of cell proliferation and inflammation, are the most important signal cascades involved in IL-6 transduction in the glomeruli and tubules of diabetic nephropathy. Thus, we determined the effects of VHH-0031 on JAK2/STAT3 pathway activation. To further confirm the suppression of JAK2/STAT3 pathway, cells were transfected with IL-6 siRNA. The results showed that after transfection of IL-6 siRNA into high-glucose-induced mesangial cells, the level of IL-6 (Figure 6A), JAK2 (Figure 6B), STAT3 (Figure 6C), TGF-β1 (Figure 6D) and Cyclin D1 (Figure 6E) significantly enhanced. Similarly, VHH-0031 treatment inhibited expression change induced by high glucose and IL-6 siRNA.

In summary, these results showed that VHH-0031 played roles in cell proliferation, ECM accumulation and inflammation induced by high glucose are depending on the IL-6R/JAK2/STAT3 pathway.

## Discussion

The number of diabetes (DM) patients has soared unprecedentedly. Currently, there are more than 400 million patients worldwide, and it is expected to reach more than 600 million by 2045 (Carracher et al., 2018). DN is the main cause of high incidence rate and mortality of ESRD, which has brought heavy medical and health care expenses to dialysis and transplantation. 20–40% of diabetic will develop DN in the course of disease (Davoud et al., 2018). In pathology, DN showed glomerular matrix expansion, basement membrane thickening and glomerular nodular sclerosis (Kimmelstiel Wilson disease). In this study, our constructed recombinant anti-IL-6R fusion proteins (VHH-0031), by linking 2 single domain chains against the pro-inflammatory cytokine receptor (IL-6R) and human serum albumin (HSA) respectively, presents potent renoprotective effect in diabetes both *in vivo* and *in vitro.*

STZ is a nitroso-containing compound, which can cause toxic effects on islet β cells in the mammalian pancreas, affecting the secretion of insulin and causing the rise of blood glucose (Wilson and Leiter, 1990). GK rat is a non-insulin-dependent and non-obese spontaneous type II diabetic rat model. The T2DM model formed by spontaneous development of GK rats is very similar to the typical Asian type diabetes. We selected STZ-induced rats and GK rats as type 1 and type 2 models *in vivo*, respectively. Then, we focused on kidney pathological changes and renal function, where the kidney is the main target organ of DN. The results showed that VHH-0031 could improve renal function and reduce the pathological severity of DN in GK rats. In STZ-induced rats, VHH-0031 could also reduce kidney damage, but it merely had a maintenance effect on blood glucose and did not make it worse. This showed that VHH-0031 exerted its renal protective effect independence on blood glucose, but displaying different effects on blood glucose levels by VHH-0031, while GK rats and STZ-induced rats showed similar improvement in kidney injury. Therefore, we speculated that VHH-0031 had the same strong anti-inflammatory effect, and could block STZ induced renal inflammation in rats, resulting in similar renal protection in both types of diabetes.

It is reported that chronic inflammation is related to the occurrence and development of DN (Navarro-González et al., 2011; Donate-Correa et al., 2015), in which cytokines are generated by various resident kidney cells, including monocytes, mesangial cells, tubular epithelial cells, podocytes and endothelial cells. IL-6 can cause glomerular basement membrane thickening by promoting the accumulation of extracellular matrix and cell proliferation (Dalla Vestra et al., 2005). In addition, it can also change endothelial permeability and expansion of mesangial cells (Navarro-González and Mora-Fernández, 2008). It is worth noting that it can amplify the inflammatory response of kidney by promoting the generation of other inflammatory cytokines, adhesion molecules and chemokines, resulting in the aggravation of DN (Sun and Kanwar, 2015). In type 1 and type 2 rat models, the level of inflammatory factors in renal tissue increased significantly, such as IL-6 and TNF-α, where VHH-0031 could significantly reduce its expression. At the same time, the level of pro-inflammatory factors increased in the mesangial cells induced by high glucose, though VHH-0031 could also inhibit the inflammatory factors. Therefore, VHH-0031 could improve renal function and reduce renal damage, which was related to its anti-inflammatory effect, rather than by exerting its insulin sensitizing activity and glucose homeostasis.

In addition to inflammatory cells and inflammatory factors, many molecules and signaling pathways are also involved in DN (Donate-Correa et al., 2020). Janus kinase (JAK) signal transducer and activator of transcription (STAT) signal pathway controls multiple biological effects such as cell proliferation, differentiation, inflammation and apoptosis. JAK is an intracellular non-receptor tyrosine kinase, which can transmit extracellular signals (including cytokines, chemokines, growth factors and hormones) after binding with membrane receptors (Marrero et al., 2006). Studies have shown that JAK2/STAT3 pathway can be activated in many cells induced by high glucose (Hu et al., 2019) and renal cortex of early DN mice (Sun et al., 2019). After ligand binding, JAK2 is activated, and then phosphorylated and STAT3 is activated. Activated STAT3 can be transported to the nucleus, binding to specific response elements in the promoter of target genes, and regulating the expression of related target genes encoding cytokines, chemokines, adhesion molecules and inducible enzymes (Li et al., 2018). The study of gene and protein expression in renal biopsy of early or late DN patients showed that JAK/STAT3 activation and expression increased (Yu et al., 2017).

All types of progressive chronic kidney disease inevitably induce renal fibrosis. In certain tissues, increased STAT3 activity mediates activation of renal interstitial fibroblasts and the progression of renal fibrosis (Pang et al., 2010). Our study confirmed that the expression of JAK2 and STAT3 increased in T1DM and T2DM rats, but inhibited by VHH-0031. Furthermore, Epithelial-mesenchymal transition (EMT) and Endothelial-mesenchymal transition (EndMT) caused by the higher levels of IL-6, IL-1β and TGF-β are the key mesenchymal inducers that play important roles in the renal fibrosis (Humphreys, 2018; Weng et al., 2019; Zhang et al., 2019). VHH-0031, which can reduce the release of IL-6, TNF-α and TGF-β, is supposed to suppress renal fibrosis induced by EndMT and EMT in diabetic kidneys so as to improve renal injury and inhibit inflammatory response. The neutrization of IL-6 by VHH-0031 is also able to rescue DN phenotype. In addition, JAK2/STAT3 could be activated in the mesangial cells treated with high glucose (Brosius and He, 2015; Heichler et al., 2020), where VHH-0031 effectively blocked the activation induced by high glucose.

Glomerular mesangial cells are one of the inherent cells of the glomerulus, which play crucial roles in maintaining renal structure and glomerular filtration rate. Glomerular mesangial cytopathy is one of the most prominent pathological changes in DN. Increasing evidence indicated that mesangial cell proliferation was characteristic of mesangial cell activation (Nahman et al., 1992). Studies showed that high glucose was one of the main factors for the occurrence of DN, and also one of the main factors to promote the proliferation of mesangial cells (Huang et al., 2013). Therefore, we choose the mesangial cells treated with high glucose as an *in vitro* model, which was consistent with other findings. In this study, high glucose promotes the release of IL-6 which can promote cycle-related genes expression, and thus enhance the cell proliferation (Ren et al., 2020). After intervention with VHH-0031, it could inhibit high-glucose-induced proliferation of mesangial cells. Flow cytometry and western blot analysis further confirmed this point. Studies had indicated that high glucose could promote JAK2 and STAT3 expression (Nahman et al., 1992). Cyclin D1 was one of the important target genes of STAT3 (Ren et al., 2020). Cell cycle disorders is an important cause of abnormal cell proliferation. In the presence of VHH-0031, HG-induced mesangial cells inhibited the activity of Cyclin D1 by down-regulating JAK2/STAT3 and blocked the cells in the G_0_/G_1_ phase and restricted their proliferation.

**FIGURE 7:**
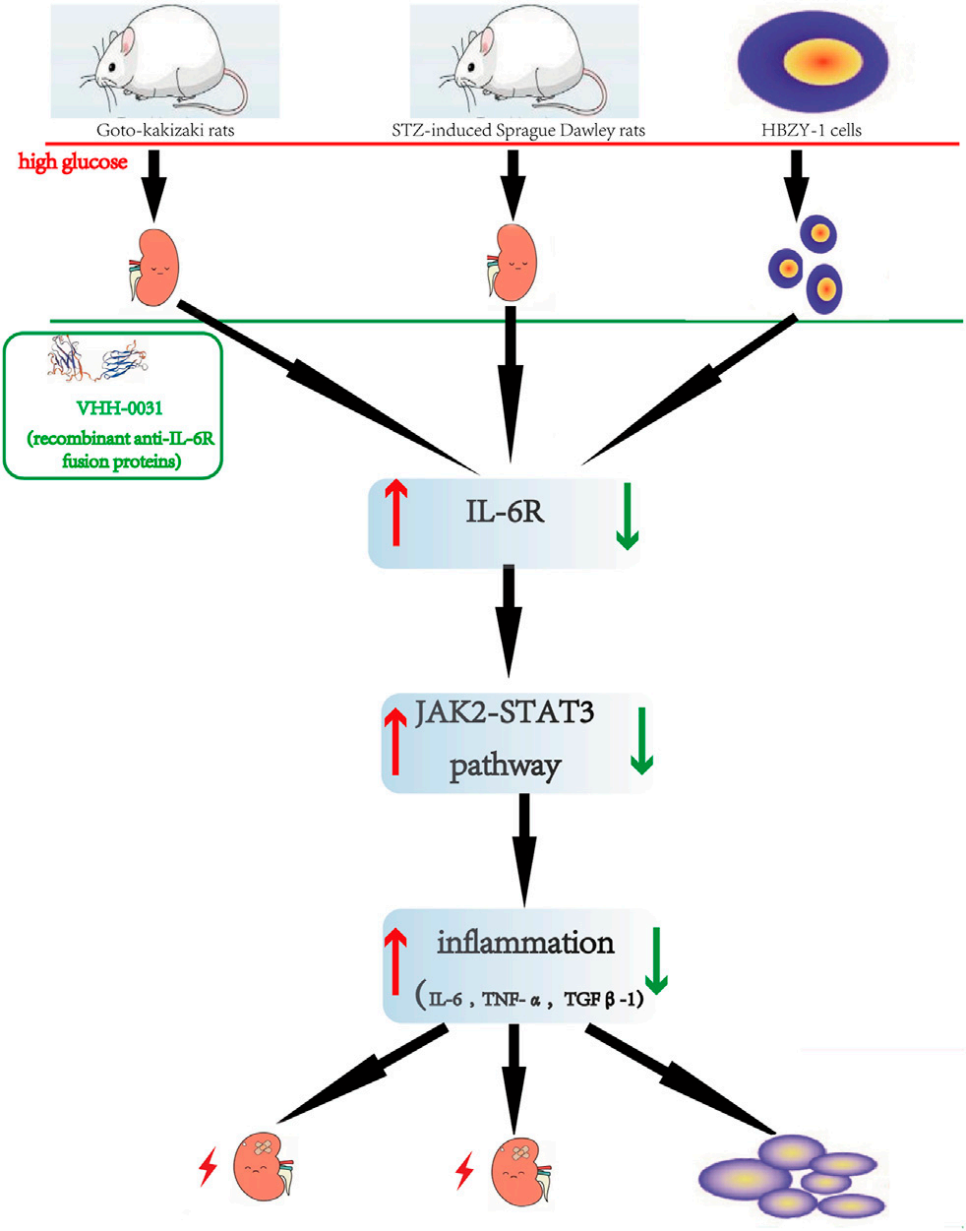
The Flowchart describes renoprotective effect of the recombinant anti-IL-6R fusion proteins (VHH-0031) by inhibiting IL6R/JAK2/STAT3 signaling pathway in diabetic nephropathy. The red of the flowchart is express that the high glucose induces kidney injury and upregulates IL6R/JAK2/STAT3 signaling pathway. After treated by anti-IL-6R fusion proteins (VHH-0031) downregulates IL6R/JAK2/STAT3 signaling pathway. The above improvement results are reflected in green.

In summary, we found that the recombinant anti-IL-6R fusion proteins (VHH-0031) through IL-6 neutrization to suppress JAK2/STAT3 signaling pathway showed a concentration dependent manner in type 1 and type 2 rats. The similar findings were further confirmed in high-glucose-induced cells *in vitro* (Figure 7). Thus, IL-6 may be a potential target to treat nephropathy caused by diabetes in clinic.

## Data Availability

The raw data supporting the conclusions of this article will be made available by the authors, without undue reservation.
